# Case Report: Personalized treatment in stage IV breast cancer using the patient-derived organoids

**DOI:** 10.3389/fonc.2025.1629690

**Published:** 2025-07-30

**Authors:** Wu Ying, Hao Pi, Xiaowu He, Yuan Sheng

**Affiliations:** ^1^ Department of Thyroid and Breast Surgery, Changhai Hospital, Naval Medical University, Shanghai, China; ^2^ Department of Breast and Thyroid Surgery, Shanghai Tenth People’s Hospital, Tongji University School of Medicine, Shanghai, China; ^3^ Department of General Surgery, The 906th Hospital of the Joint Logistic Support Force of the Chinese People’s Liberation Army, Ningbo, China

**Keywords:** stage IV breast cancer, patient-derived organoids, sacituzumab govitecan, neoadjuvant therapy, pathological response

## Abstract

Breast cancer, especially stage IV breast cancer, is highly heterogeneous at morphological and molecular levels. Recently, the role of patient-derived organoids (PDOs) has become increasingly prominent in cancer research and personalized medicine. They are not only used to predict the clinical responses of patients with multiple cancer types, but also applied to drug development. In this study, we reported a case of stage IV breast cancer who responded well to sacituzumab govitecan that was highly sensitive by the PDO-based drug sensitivity testing, good pathological response was obtained postoperatively. This typical case suggests that PDO-based drug sensitivity testing is conductive to tailoring neoadjuvant chemotherapy options to enhance the feasibility of surgical resection in patients with stage IV breast cancer, thus improving the prognosis.

## 1 Introduction

Breast cancer is one of the most common malignancies in women worldwide, with high incidence and mortality (1). As a molecularly heterogeneous disease, the clinical outcomes of breast cancer mainly depend on its biological subtypes (2). Human epidermal growth factor receptor 2 (HER2)-targeted therapy, endocrine therapy and systemic chemotherapy are commonly used treatment modalities for breast cancer, but there still exist a portion of patients who exhibit resistance to the initial therapy and eventually experience recurrence (3). Neoadjuvant chemotherapy (NAC), a novel treatment model, can not only decrease the tumor stage, increase the conservative operation rate, but also help to identify the unresponsive tumors (4). It is reported that by comparison to those with residual diseases, breast cancer patients achieving complete pathological response (pCR) after NAC show better survival outcomes (5). Nevertheless, it is unknown about whether the breast cancer patients receiving NAC can obtain pCR. Additionally, it is also difficult to assess the response to monotherapy for the patients treated with combined therapy at the individual level.

Patient-derived organoids (PDOs), a newly developed three-dimensional cell culture technology, can not only preserve the complicated spatial architecture and cellular heterogeneity of parental tumors, but also recapitulate their morphological, genetic and molecular features (6). In recent years, PDOs have emerged as a promising model system for cancer research and personalized medicine, and there shows a strong association between PDOs and clinical outcomes in cancer regarding the prediction of response to various therapies including chemotherapy, targeted therapy and radiotherapy (7–9). Here, we shared a case of stage IV breast cancer whose NAC regimens were tailored based on the PDO drug sensitivity testing, and good pathological response was achieved after modified radical mastectomy.

## 2 Case presentation

**Figure 1:**
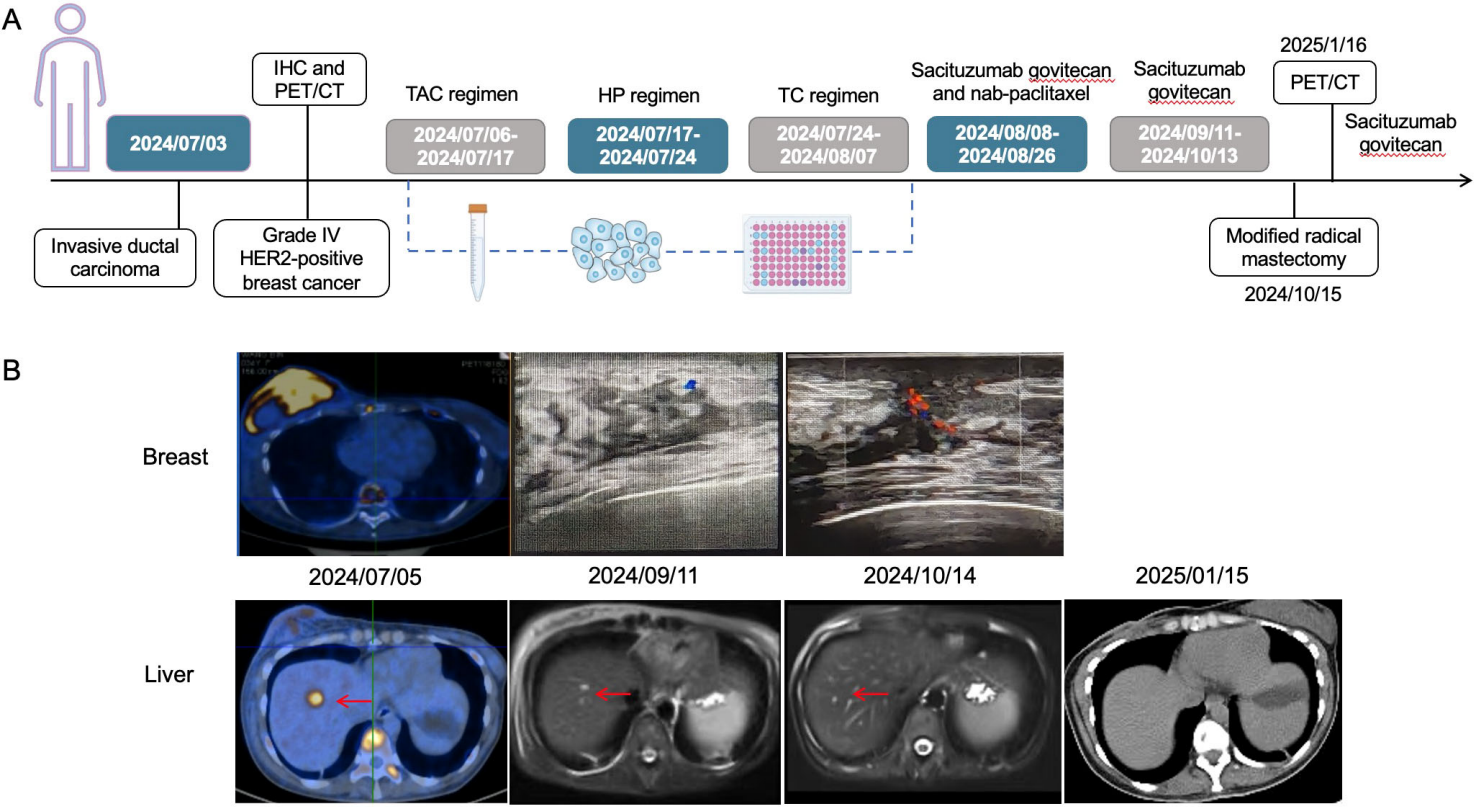
The timeline of diagnosis and treatment process of the patient **(A)**, and radiological variations in breast and liver metastatic lesions during the treatment **(B)**. The red arrows head toward the lesions. It could be observed that the liver metastatic lesions were gradually reduced until disappearance as time went by. TAC regimen: cyclophosphamide, doxorubicin and docetaxel; HP regimen: trastuzumab and pertuzumab; TC regimen: docetaxel and cyclophosphamide.

A 35-year-old woman presented to a hospital with the chief compliant of a gradually increased mass in the right breast for 2 months. She previously had no history of drinking, smoking, infectious disease, allergy and surgical resection. On July 3, 2024, she underwent a needle biopsy for the mass in the right breast and lymph nodes in the right armpit under local anesthesia. The pathological results showed grade II invasive ductal carcinoma, accompanied by tumor metastases with the maximum diameter of 6 mm. Immunohistochemical results showed ER (-), PR (-), HER2 (3+) and Ki-67 (20%), and HER2 amplification was confirmed by FISH. Physical examinations showed a huge, hard mass in the right breast, with multiple cancerous satellite lesions and the necrotic nipple and areolae. In the right armpit, a 2-cm nodule was touched. Positron emission tomography/computed tomography imaging (PET-CT) revealed right breast cancer invading nipples and skin, accompanied by multiple lymphatic metastases in the neck, mediastina, bilateral armpits, interpectoral spaces and lung hilus, multiple metastases in the liver as well as bone metastasis (**Figure 1**). No mutations in *PIK3CA* including H1047R, H1047L, E542K, E545K and E545D were identified in the tissue from the right armpit. In combination of relevant examinations, the patient was diagnosed with stage IV (T4N3M1) HER2-positive breast cancer. The levels of tumor markers were listed in **Table 1**.

**Table 1:** Changes of several important tumor markers in the patient before and after organoid-guided treatment.

Variables	CEA (ng/mL)	CA125 (U/mL)	CA153 (U/mL)	CA199 (U/mL)
On admission	35.7	25.2	125	102
Before organoid-based NAC	23.0	19.2	138.7	42.9
After 1-cycle of organoid-guided NAC	7.9	16.2	63.4	18.2
After 2-cycle of organoid-guided NAC	4.7	12.0	44.2	14.7
After modified radical mastectomy	1.9	11.4	32.5	13.8
5 months after modified radical mastectomy	4.1	14.3	31.9	19.5

NAC, neoadjuvant chemotherapy; CEA, carcinoembryonic antigen; CA, carbohydrate antigen.

On July 8, 2024, the patient underwent rotary-cut biopsy for the right mass. After fully informing the patient and family members of the possible consequences and benefits, they agreed to receive precision testing for guiding the treatment. Therefore, a portion of biopsy sample was transported to the laboratory on ice for organoid culture. PDO establishment and drug sensitivity testing were conducted strictly according to the protocols provided by Kingbio Medical (Chongqing) Co., Ltd. Specifically, the tumor tissues were cut into pieces using sterile scissors and then were digested with Jiabili^®^ tissue digestive juice for 30 min. After digestion, the supernatant was absorbed to another centrifuge tube of 15 mL for temporary storage on ice. When digestion terminated, 3-5 mL Jiabili^®^ tumor tissue basic medium II was added to wash 3 times, and 1 mL pipette tips were used to vigorously blow up and down 10-20 times. Subsequently, the supernatant was centrifuged, and sedimentation was collected. According to the amount of sedimentation, Matrigel was added. After mixing, the cells and Matrigel suspension were seeded onto multi-well plates. Once the droplets were solidified, appropriate amount of Jiabili^®^ organoid medium for breast cancer was added. Finally, the cell status and aggregation in Matrigel were observed microscopically, the plates were placed in a 37°C, 5%CO_2_ incubator for culture. The culture medium was replaced every 2-3 days until the organoids grew like solid spheroids with the diameter of about 70 µm. Regarding the drug sensitivity testing, Jiabili^®^ organoid digestive juice was first added to the established organoids until the organoid cell masses were digested into single cells. Then, appropriate amount of Jiabili^®^ tumor tissue basic medium II was used for resuspension, and the organoid number was calculated. When Jiabili^®^ organoid medium for breast cancer and Matrigel were added, the suspension seeded onto multi-well plates was placed in a 37°C incubator. Subsequently, the pre-prepared drug solution and CelltiterGlo 2.0 were added, respectively. Finally, the drug sensitivity testing results were analyzed by detecting the total number of viable cells.

**Figure 2:**
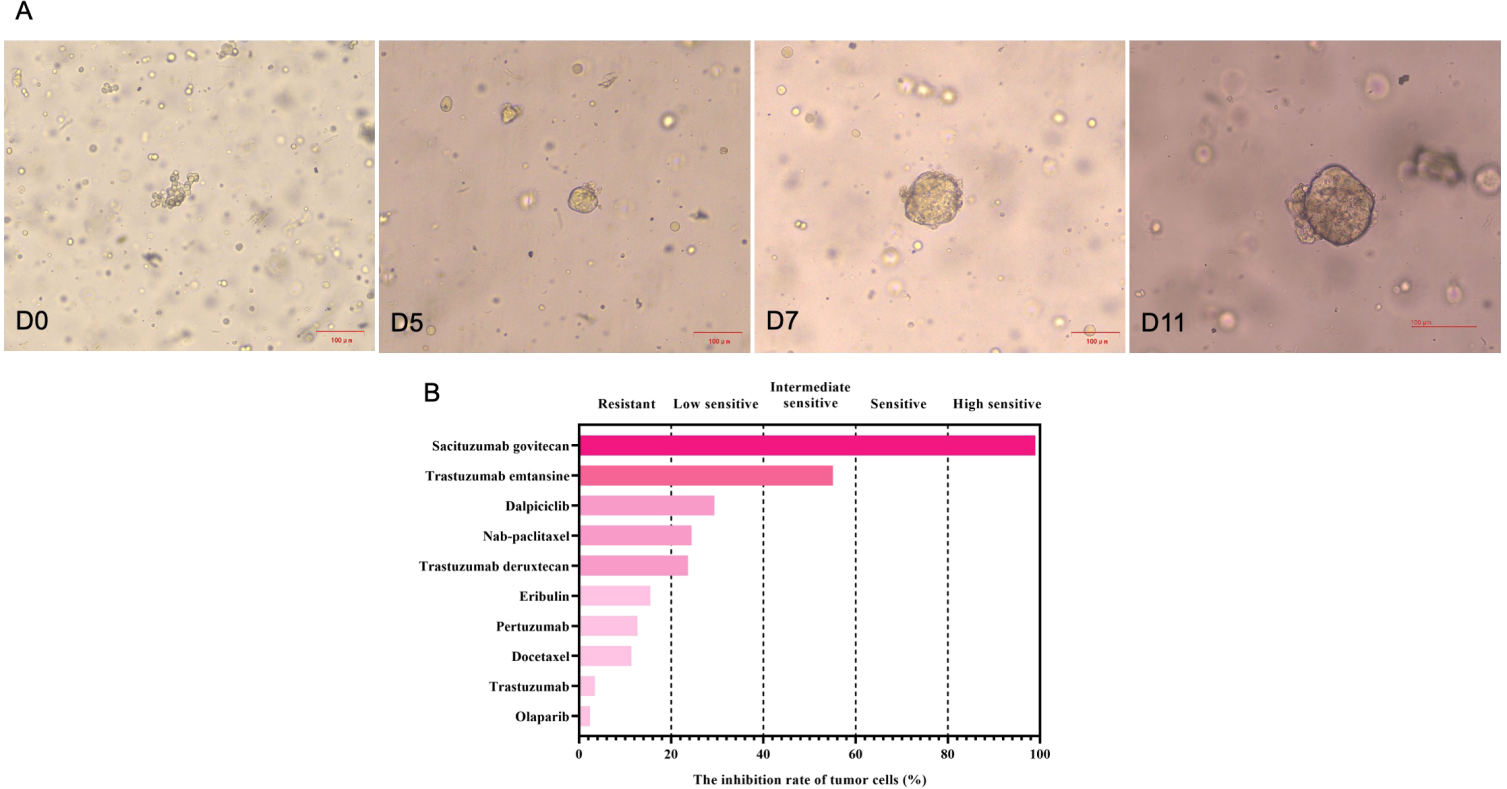
The growth changes of the stage IV HER2-positive breast cancer organoids from biopsy samples at different time points **(A)**, and drug sensitivity testing results **(B)**. Based on the inhibition of tumor growth rates, five categories were used in this study, including highly sensitive (>80%), sensitive (60%-80%), intermediate sensitive (40%-60%), lowly sensitive (20%-40%) and resistant (<20%).

Through drug sensitivity testing, it could be observed that the patient was highly sensitive to sacituzumab govitecan (**Figure 2**). During organoid culture, the patient was first treated with one cycle of TAC regimen (cyclophosphamide, doxorubicin and docetaxel). Subsequently, neoadjuvant chemotherapy was adjusted to HP (trastuzumab and pertuzumab) and TC (docetaxel and cyclophosphamide) regimens, respectively. On August 8, 2024, sacituzumab govitecan (360 mg, d1, 8) combined with nab-paclitaxel (200 mg, d1, 8) was administrated based on organoid drug screening results. On September 11, 2024, ultrasound B examinations for the breasts showed multiple diffuse low-echo areas in the right breast, with partial fusions; the larger one (35×22 mm) was in the outer upper quadrant, with unclear boundary and irregular morphology. Meanwhile, there was also a lymphatic echo (8×4 mm) in the right armpit (**Figure 1**). Enhanced liver MRI revealed multiple liver metastases, with the largest one of 7 mm (**Figure 1**). The levels of tumor markers were decreased (**Table 1**). Due to intolerable adverse reactions including nausea, vomiting, and abdominal pain, sacituzumab govitecan was administrated alone for further treatment after discussion with the patient. On October 14, 2024, the breast ultrasound B examination showed that the low-echo area in the outer upper quadrant (33×18 mm) and lymphatic echo (6×5 mm) in the right armpit were both reduced, and liver metastases were also significantly decreased than before, with the largest one of 3 mm (**Figure 1**). Considering that the patient and family members requested surgery, and the patient’s various indicators were tested to meet the surgical requirements. October 15, 2024, modified radical mastectomy for right breast cancer was performed. Postoperative pathology showed lobular atrophy and interstitial fibrosis of breast tissues in the right primary breast tumor bed, accompanied by mass-like aggregation of many foam-like histiocytes and lymphocytes, multifocal necrosis and calcification. Additionally, multiple scattered ductal carcinomas *in situ* were also seen, without invasive cancer cells, which was consistent with a small quantity of tumor residues [Miller-Paney grade 5, residual cancer burden (RCB) I] after neoadjuvant treatment. Postoperatively, sacituzumab govitecan was used continuously. On January 16, 2025, the PET-CT indicated slightly incrassated soft tissues in the right chest wall, multiple bone metastases, and pathological fractures of the chest vertebral body 8, without significant abnormalities in the brain. The CT scan showed that the metastatic lesions in the liver almost disappeared completely (**Figure 1**). Up to now, the tumor marker levels of the patient remained stable (**Table 1**).

## 3 Discussion

For appropriately selected patients with de nova stage IV breast cancer, primary tumor resection not only limits locoregional progression, but also contributes to improving survival outcomes (10). Nevertheless, practitioners should comprehensively consider the patients’ age, tumor types, performance status, comorbidities and metastatic disease burden to determine the necessity of primary tumor resection. For breast cancer patients meeting the NAC indications, multidrug combinations or NP sequential therapy are usually applied when NAC is performed. In clinical practice, however, it is difficult to assess the sensitivity of the single drug for patients receiving combined therapy at the individual level. As a promising tool for personalized medicine in cancer, PDOs have been confirmed to be a superior model for drug screening and prediction of treatment response (7, 11). In this study, we successfully established breast cancer organoids from biopsy samples and demonstrated that PDOs could be used to tailor the NAC options to promote the feasibility of surgical resection in stage IV breast cancer by screening the sensitivity of the single drug or combined drugs, consequently leading to prognostic improvement.

Approximately 20%-25% of breast cancer patients experience HER2 amplification, which has been confirmed to be associated with poor prognosis and can be a therapeutic target (12). Recently, dual-target therapy for HER2-positive breast cancer has been routinely recommended in clinical practice, showing good results. Trastuzumab and pertuzumab are the two commonly used HER2 monoclonal antibodies. For the HER2-positive patient in our study, we adopted trastuzumab and pertuzumab as the NAC for treatment after use of TAC regimen for one cycle, and then adjusted into TC regimen for further treatment due to unsatisfactory therapeutic effects until the presence of PDO drug sensitivity testing results. It could be observed that the patient was highly sensitive to the targeted drug sacituzumab govitecan and relatively sensitive to nab-paclitaxel compared with other chemotherapy drugs, thus sacituzumab govitecan combined with nab-paclitaxel was used. After two cycles, significant reductions were identified whether in the primary tumor or metastatic lesions, and better pathological response was achieved following modified radical mastectomy. Sacituzumab govitecan, a first-in-class trophoblast cell-surface antigen 2 (Trop-2)-directed antibody and topoisomerase I inhibitor conjugate, has been globally approved for treating the adults with unresectable locally advanced or metastatic, hormone receptor-positive/HER 2-negative breast cancer (13), and speedily approved for the adults with metastatic tiple-negative breast cancer who previously received at least two therapies for metastatic diseases (14, 15). Notably, the patient in our study was diagnosed with metastatic, HER2-positive breast cancer, and achieved good responses to sacituzumab govitecan, which might be partially explained by the tumor heterogeneity. Currently, there are no data from clinical trials on sacituzumab govitecan alone or its combination with HER2-targeted agents for HER2-positive breast cancer. According to previous literature, Trop-2 is broadly expressed in all breast cancer subtypes (16). In the subgroup analysis of TROPiCS-02 trial, significantly prolonged progression-free survival was observed in HER2-low breast cancer patients receiving sacituzumab govitecan, suggesting Trop-2-targeted therapy may be effective to HER2-low or heterogeneous tumors (17). As a Trop-2-targeted antibody-drug conjugate, sacituzumab govitecan has been gradually incorporated as a part of the standard regimens for all subtypes of advanced breast cancer, as well as for high-risk HER2-positive breast cancer (18).

Breast cancer, especially stage IV breast cancer, is highly heterogeneous at morphological and molecular levels. Metastatic tumor cells can invade into different organs, and complex tumor heterogeneity, plasticity and tumor microenvironment can influence the therapeutic response collectively and shape resistance (19). Currently, empirical anti-tumor therapy may not be able to maximize the benefits of such patients. The advent of precision medicine brings hope for the individualized treatment of cancer patients. In recent years, the role of PDOs in cancer research and precision medicine has been increasingly prominent. They are not only used to predict the clinical responses of patients with multiple cancer types, but also applied to drug development. Through optimization of treatment regimens and minimization of unnecessary side effects, organoid-based drug sensitivity testing show promise in improving the treatment outcomes of patients at the individual level (20). In a recent study, the clinical outcomes of patients with advanced breast cancer receiving organoid-guided therapy were compared with those receiving treatment of physician’s choice, and the results indicated that organoid-guided therapy was associated with extended progression-free survival and improved disease control rate, highlighting the importance of PDOs in optimizing the breast cancer treatment regimens via precision medicine strategies (21). Clinical case studies from previous literature further confirmed the survival benefits in advanced breast cancer patients treated with organoid-guided therapy (22). In our study, the patient experienced liver metastases, bone metastases, and involvement of the entire breast at initial diagnosis, indicating a heavy tumor burden. Therefore, the treatment regimens including TAC, HP and TC were successively used, but the efficacy was unsatisfactory. Based on the organoid-guided treatment, modified radical mastectomy was successfully performed, and good postoperative pathological response was obtained. Although bone metastases still existed postoperatively, liver metastatic lesions disappeared and no local recurrence was observed in the breast lesions, further indicating that organoid-guided treatment was effective. However, some limitations should be noted regarding the application of PDOs. First, culture proto cols should be standardized to ameliorate organoid repeatability and reproducibility. Second, tumor organoid models are lack of *in vivo* components, such as immune cells, fibroblasts and endothelial cells. Recently, the organoids cocultured with immune and vascular cells have attempted to be established, and this restriction will be resolved shortly.

In conclusion, we described a stage IV breast cancer patient responding well to sacituzumab govitecan that was highly sensitive by the PDO-based drug sensitivity testing, good pathological response was obtained. This typical case suggests that PDO-based drug sensitivity testing is conductive to tailoring NAC options to enhance the feasibility of surgical resection in patients with stage IV breast cancer, thus improving the prognosis.

## Data Availability

The original contributions presented in the study are included in the article/Supplementary Material. Further inquiries can be directed to the corresponding author.
